# Zebrafish models of COVID-19

**DOI:** 10.1093/femsre/fuac042

**Published:** 2022-11-02

**Authors:** Sylwia D Tyrkalska, Sergio Candel, Annamaria Pedoto, Diana García-Moreno, Francisca Alcaraz-Pérez, Álvaro Sánchez-Ferrer, María L Cayuela, Victoriano Mulero

**Affiliations:** Departmento de Biología Celular e Histología, Facultad de Biología, Universidad de Murcia, 30100 Murcia, Spain; Instituto Murciano de Investigación Biosanitaria (IMIB)-Arrixaca, 30120 Murcia, Spain; Centro de Investigación Biomédica en Red de Enfermedades Raras (CIBERER), Instituto de Salud Carlos III, 28029 Madrid, Spain; Departmento de Biología Celular e Histología, Facultad de Biología, Universidad de Murcia, 30100 Murcia, Spain; Instituto Murciano de Investigación Biosanitaria (IMIB)-Arrixaca, 30120 Murcia, Spain; Centro de Investigación Biomédica en Red de Enfermedades Raras (CIBERER), Instituto de Salud Carlos III, 28029 Madrid, Spain; Departmento de Biología Celular e Histología, Facultad de Biología, Universidad de Murcia, 30100 Murcia, Spain; Instituto Murciano de Investigación Biosanitaria (IMIB)-Arrixaca, 30120 Murcia, Spain; Centro de Investigación Biomédica en Red de Enfermedades Raras (CIBERER), Instituto de Salud Carlos III, 28029 Madrid, Spain; Instituto Murciano de Investigación Biosanitaria (IMIB)-Arrixaca, 30120 Murcia, Spain; Centro de Investigación Biomédica en Red de Enfermedades Raras (CIBERER), Instituto de Salud Carlos III, 28029 Madrid, Spain; Instituto Murciano de Investigación Biosanitaria (IMIB)-Arrixaca, 30120 Murcia, Spain; Centro de Investigación Biomédica en Red de Enfermedades Raras (CIBERER), Instituto de Salud Carlos III, 28029 Madrid, Spain; Grupo de Telomerasa, Cáncer y Envejecimiento (TCAG), Hospital Clínico Universitario Virgen de la Arrixaca, 30120 Murcia, Spain; Instituto Murciano de Investigación Biosanitaria (IMIB)-Arrixaca, 30120 Murcia, Spain; Departmento de Bioloquímica y Biología Molecular A, Facultad de Biología, Universidad de Murcia, 30100 Murcia, Spain; Instituto Murciano de Investigación Biosanitaria (IMIB)-Arrixaca, 30120 Murcia, Spain; Centro de Investigación Biomédica en Red de Enfermedades Raras (CIBERER), Instituto de Salud Carlos III, 28029 Madrid, Spain; Grupo de Telomerasa, Cáncer y Envejecimiento (TCAG), Hospital Clínico Universitario Virgen de la Arrixaca, 30120 Murcia, Spain; Departmento de Biología Celular e Histología, Facultad de Biología, Universidad de Murcia, 30100 Murcia, Spain; Instituto Murciano de Investigación Biosanitaria (IMIB)-Arrixaca, 30120 Murcia, Spain; Centro de Investigación Biomédica en Red de Enfermedades Raras (CIBERER), Instituto de Salud Carlos III, 28029 Madrid, Spain

**Keywords:** SARS-CoV-2, animal models, senescence, telomeres, drug repurposing, zebrafish

## Abstract

Although COVID-19 has only recently appeared, research studies have already developed and implemented many animal models for deciphering the secrets of the disease and provided insights into the biology of SARS-CoV-2. However, there are several major factors that complicate the study of this virus in model organisms, such as the poor infectivity of clinical isolates of SARS-CoV-2 in some model species, and the absence of persistent infection, immunopathology, severe acute respiratory distress syndrome, and, in general, all the systemic complications which characterize COVID-19 clinically. Another important limitation is that SARS-CoV-2 mainly causes severe COVID-19 in older people with comorbidities, which represents a serious problem when attempting to use young and immunologically naïve laboratory animals in COVID-19 testing. We review here the main animal models developed so far to study COVID-19 and the unique advantages of the zebrafish model that may help to contribute to understand this disease, in particular to the identification and repurposing of drugs to treat COVID-19, to reveal the mechanism of action and side-effects of Spike-based vaccines, and to decipher the high susceptibility of aged people to COVID-19.

## Teaser

We review the unique advantages of the zebrafish model that may help to the identification and repurposing of drugs to treat COVID-19, to reveal the mechanism of action and side-effects of Spike-based vaccines, and to decipher the role of aging in COVID-19.

## Introduction

Throughout history there have been several pandemics of human diseases, that have emerged in different geographical areas and have been caused by numerous pathogens that were able to cross the species barrier to infect humans and cause human-human transmission. The most recent pandemic of Coronavirus disease 2019 (COVID-19) was declared on 11th of March 2020 by the World Health Organization, following the identification of a novel coronavirus strain called severe acute respiratory syndrome coronavirus 2 (SARS-CoV-2) (Gralinski and Menachery 2020, Viruses 2020). Its initial genomic sequencing did not match those of previously sequenced coronaviruses. For the first time the virus was identified in December 2019 in the city of Wuhan (China) from where the virus spread uncontrollably all over the world becoming a serious global health threat and leading to widespread social and economic disruption (Huang et al. 2020). To date, it has been detected in over 220 countries with over 600 million positive cases and over 6 million deaths (WorldDoMeter 2022).

Although several animal models have been implemented for deciphering the secrets of the disease, there are several major factors that complicate the study of this virus, such as the poor infectivity of clinical isolates of SARS-CoV-2 in some model species and the absence of the systemic complications which characterize COVID-19 clinically (Muñoz-Fontela et al. 2020). Another important limitation is the difficulties to model the impact of aging in COVID-19. Therefore, we review here the main animal models developed so far to study COVID-19 and introduce the unique advantages of the zebrafish model for the identification and repurposing of drugs to treat this disease, to reveal the mechanism of action and side-effects of Spike-based vaccines, and to decipher the high susceptibility of aged people to COVID-19.

### SARS-CoV-2 Virus Structure

Taxonomically, SARS-CoV-2 belongs to the *Betacoronavirus* genus and is a member of the *Coronavirinae* family, *Nidovirales* order and *Riboviria* realm (Mittal et al. 2020, Rehman et al. 2020). It possesses an unsegmented, single-stranded, positive-sense RNA genome of 29891 bp in length, with a G + C content of 38%, which is enclosed by a 5′-cap and 3′-poly(A) tail (Fehr and Perlman 2015, Chan et al. 2020a). SARS-CoV-2 presents similar aspects to other members of the coronavirus family regarding its genome, its identity reaching 79% with SARS-CoV and 50% with MERS-CoV, and its protein structure and infection mechanisms (Lu et al. 2020a). SARS-CoV-2 genome is arranged linearly and is composed of 10 open reading frames (ORF) of which 67% encodes for nonstructural polyproteins and the remaining 33% for accessory and structural proteins (Mittal et al. 2020). The virus particles are spherical or pleomorphic in shape, with a diameter of about 60-140 nm (Zhu et al. 2020). Single virions are encircled with an envelope containing viral nucleocapsid with distinct spikes of 9-12 nm, giving the virus the appearance of solar corona (Zhu et al. 2020).

**Figure 1.**
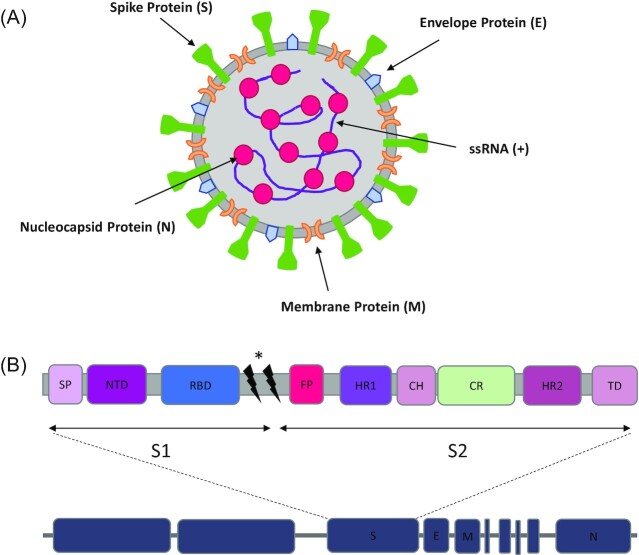
SARS-CoV-2 structure. **(A)** Structure of single virion of SARS-CoV-2 including structural proteins S, M, E, and N as well as the single stranded RNA. **(B)** Schematic representation of the genomic structure of SARS-CoV-2 genome with individual components of S protein (not to scale). E—envelope protein gene; M—membrane protein gene; N—nucleocapsid protein gene, S—spike protein gene; SP—signal peptide; NTD—N-terminal domain; RBD—receptor binding domain; FP—fusion protein; HR1- heptad repeat 1; CH—central helix; CR—connecting region; HR2–heptad repeat 2; TD—transmembrane domain; * protease cleavage sites (including furin cleavage site).

Among the proteins expressed by the SARS-CoV-2 genome, four major structural proteins can be distinguished: the spike (S), membrane (M), envelope (E) and nucleocapsid (N) proteins (Fig. 1) (Srinivasan et al. 2020). S protein consists of a membrane-distal S1 subunit and a membrane-proximal S2 subunit, forming homotrimers in the virus envelope. The S1 subunit is mainly responsible for recognition of the host cellular receptor via its receptor-binding domain (RBD) located at the C-terminal, whereas N-terminal of S1 subunit is composed of N-terminal domain (NTD). The S2 subunit determines membrane fusion which is necessary to initiate virus entry (Beniac et al. 2006, Belouzard et al. 2012). The S2 subunit possesses fusion peptide (FP), connecting region (CR), heptad repeat 1 (HR1), and heptad repeat 2 (HR2) situated around a central helix (Fig. 1). Structural rearrangement of SARS-CoV-2 S protein has been proposed following recognition by the host cell receptor (Cai et al. 2020). Proteolytical cleavage of S protein can occur at two cleavage sites, separating S1 and S2 and releasing the structural constrains of the mature FP (Zamorano Cuervo and Grandvaux 2020, Dai and Gao 2021). The surface of S protein is extensively shielded by glycans, except RBD, a quality that may facilitate immune escape of the virus.

The RBD of the Spike binds to the host receptor, human angiotensin-converting enzyme 2 (hACE2), through a specific receptor-binding motif (RBM) situated on its external subdomain. This ligand-receptor binding leads to the dissociation of the S1 subunit and simultaneously initiates the refolding process of the spring-loaded S2 subunit, triggering membrane fusion via FP, allowing HR1 and HR2 to refold, and forming a post-fusion conformation. Subsequently, the S2 subunit in its post-fusion conformation folds as a long helical bundle with the FP inserted into the host cell membrane (Dai and Gao 2021).

M proteins are embedded in the envelope, giving shape to the virion wrapper. They are present in high amounts out of all coronavirus proteins consisting of an N-terminal domain attached to the triple transmembrane domains, which are further connected to a carboxyl-terminal domain (Arndt et al. 2010, J Alsaadi and Jones 2019). This interaction helps to encapsulate the SARS-CoV-2 RNA genome. It has recently been recognized that M proteins may regulate the host immune response and viral pathogenicity (Zhang et al. 2021, Yang et al. 2022, Zambalde et al. 2022). E proteins are tiny integral membrane polypeptides that are crucial for coronavirus infectivity. They are composed of NTD, hydrophobic domain and a chain at C-terminal. The hydrophobic region oligomerizes to form a ionic pore across the membrane (Ruch and Machamer 2011). Finally, N proteins make up the helical nucleocapsid and bind along the viral RNA genome to form a core of a ribonucleoprotein, which helps in its host cell entry and interact with cellular processes following the fusion of virus (Huang et al. 2004). They are already expressed in the host during the early stages of SARS-CoV-2 infection.

Besides structural proteins, SARS-CoV-2 encodes non-structural proteins and accessory proteins. It has been shown that the first two thirds of the coronavirus genome encodes replicase genes, which are translated into two large polyproteins, pp1a and pp1ab, that are processed into 15 non-structural proteins via proteolytic cleavage by virus encoded proteases (nsp1-10 and nsp12-16) (Thiel et al. 2003, Wu et al. 2020). Coronavirus genome also contains a variable number of ORFs coding for accessory proteins that are not essential for its replication or structure but appear to have a role in pathogenesis in the natural host (Liu et al. 2014). These genes are seen to be intermingled with the structural genes. SARS-CoV-2 possesses eight accessory genes: 3a, 3b, 6, 7a, 7b, 8, 9b, and 10, and lacks the hemagglutinin-esterase gene (HE) that is present in SARS-CoV (Fehr and Perlman 2015, Chan et al. 2020a).

RNA viruses have much higher mutation rates within their genome comparing with DNA viruses, which in numbers means about one mutation per virus genome copy. SARS-CoV-2 genome is susceptible to mutations during the replication process due to the errors generated by RNA-dependent RNA polymerases (Lau et al. 2021), despite coronavirus RNA-dependent RNA polymerases have proofreading activity and thus they mutate less than most RNA viruses (Robson et al. 2020). Adaptive mutations may provoke alterations in the virus's pathogenic potential, where even a single amino acid exchange can drastically affect or even perturb its ability to evade the host immune system and hence complicate the progression of vaccine development against the virus. Genetic evolution caused by development of mutations in viral genome results in the emergence of multiple variants of the same virus that may have different characteristics compared to their ancestral strains (Tao et al. 2021). Four SARS-CoV-2 variants of concerns (VOCs) have evolved to date: Beta (lineage B.1.351), Gamma (lineage P.1), Delta (lineage B.1.617.2) and Omicron (lineage B.1.1.529) (Duong 2021). The common feature of all SARS-CoV-2 VOCs is the possession of new and at the same time different mutations mostly in the region of Spike protein changing its transmissibility and infectivity. Their main characteristics of the VOCs are:

- Beta: detected in South Africa in the late 2020, shows three mutations in RBD of S protein: N501Y, K417N, and E484K, that facilitate its attachment to the host cell and hence increase its transmissibility and disease severity (Duong 2021).
- Gamma: this variant arose in Brazil in the beginning of 2021 with ten new mutations within Spike protein including the three mutations of most concern, N501Y, E484K and K417T affecting the binding affinity between RBD and ACE2 and increasing its transmissibility, and producing ten-fold increase in viral load in patients (Duong 2021).
- Delta: was discovered in the late 2020 in India and possesses substitutions in T478K, L452R and P681R within S protein, which increase its transmissibility and facilitate its immunoescape (Duong 2021).
- Omicron: detected in Botswana in the late 2021 and possessing 32 new mutations affecting Spike protein, hence being able to multiply 70 times much faster than Delta variant. However, it has lower ability to penetrate the lung tissue, lower fatality rate and risk of hospitalization (Karim and Karim 2021).

### SARS-CoV-2 Virus Integration, Cellular Interaction, and Signaling Pathways Activation

Human ACE2 is a type-I transmembrane receptor and a zinc-containing metalloenzyme that is mostly located on the cell surface of different tissues (Gheblawi et al. 2020). It contains N-terminal peptidase M2 domain and C-terminal collectrin renal amino acid transporter domain (Gheblawi et al. 2020). The receptor ACE2 is widely expressed in the human body, including many different systems such as renal, lymphoid, cardiovascular, gastrointestinal, respiratory and central nervous (Harmer et al. 2002). More specifically, ACE2 has been identified in alveolar epithelium cells and capillary endothelium of the lungs, small intestine epithelia, blood vessels and capillaries of the skin, brain endothelium and renal glomerular epithelium (Hamming et al. 2004). Given the detection of ACE2 protein in organs that are targets for SARS-CoV-2 and the mechanisms associating ACE2 with the invasion and replication of SARS-CoV, ACE2 serves as a functional receptor for SARS-CoV-2. The α1 and α2 helixes and the loop that connect β3 and β4 antiparallel strands are mediating the interaction (Yan et al. 2020).

It has been demonstrated that the entry process of the SARS-CoV-2 virus into the host cell is mediated by high affinity binding of the S protein to ACE2, followed by the processing of transmembrane serine protease 2 (TMPRSS2). This enables the priming of S protein to promote membrane fusion and SARS-CoV-2 entry into the host cells (Hoffmann et al. 2020). The co-expression of both proteins, TMPRSSs and ACE2, is a crucial factor that determines the entry of SARS-CoV-2 into host cells (Gheblawi et al. 2020, Hoffmann et al. 2020, Walls et al. 2020). Interestingly, the S protein from the SARS-CoV-2 binds to ACE2 with about a ten-fold higher affinity than SARS-CoV, which facilitates virus invasion and its spreading to different tissues (Wrapp et al. 2020). Additionally, it has been shown that for ACE2 processing, TMPRSS2 competes with a disintegrin and metalloproteinase domain-containing protein 17 (ADAM17). However, only cleavage by TMPRSS2 appears to promote S protein-driven cellular entry (Heurich et al. 2014). Endosomal cathepsin can also process S protein of SARS-CoV-2 in cell lacking TMPRSS activity (Jackson et al. 2022). However, it has been recently reported that the release of the virion into the cytoplasm required acid pH and occurs principally from endosomes irrespective of the virus dependence of TMPRSS2 or cathepsin.

Moreover, ACE2 expression has been shown to highly correlate with the expression of alanyl aminopeptidase (ANPEP) and dipeptidyl peptidase-4 (DPP4), known receptors for other human coronaviruses (Raj et al. 2013, Li 2015, Peck et al. 2017). This seems to suggest that these proteins act as co-receptors or auxiliary SARS-CoV-2 receptors in the process (Qi et al. 2020). Furthermore, the identification of transmembrane glycoprotein CD147 as a novel protein taking part in the virus entry route (Wang et al. 2020a), the presence of furin-like cleavage sites in the S protein that are absent for other SARS-CoVs (Coutard et al. 2020) and the stabilization as well as destabilization of viral gene mutations, endosome-associated-protein-like domain of nsp2 protein and phosphatase domain of nsp3, respectively (Angeletti et al. 2020), might be closely related to viral–host mechanisms of invasion and play a role in the life cycle of the virus and disease pathogenicity, resulting in the particularly contagious nature of SARS-CoV-2.

Activation of the ACE2 receptor, a key enzyme in the Renin–Angiotensin system (RAS), plays a physiological role in regulating renal–cardiovascular systems and the innate immunity (Zhang et al. 2014, Cao et al. 2020). In this pathway, the kidney produces renin that cleaves Angiotensinogen from the liver, producing Angiotensin (Ang)-I, which then is cleaved by ACE2 into Angiotensin (Ang)-II. The latter is able to bind to the Angiotensin II type 1 receptor (AT1R) and Angiotensin II type 2 receptor (AT2R) executing their functions (Zhang et al. 2014, Paz Ocaranza et al. 2020). Because of the high affinity of SARS-CoV-2 S protein towards hACE2, the RAS system appears to have a central role in SARS-CoV-2 infection.

SARS-CoV-2 infection may cause ACE2 down-regulation in many tissues (Gheblawi et al. 2020), and may be up-regulated by interferon (IFN). However, the relevance of ACE2 upregulation by IFNs is controversial, since some reports found that IFNs only upregulate its truncated isoform (Onabajo et al. 2020, Scagnolari et al. 2021). Unfortunately, ACE2 inhibition does not have a beneficial effect during SARS-CoV-2 infection, since it leads to a more extensive conversion of Ang-I into Ang-II via ACE, and its binding to ATR1 receptors to further promote vascular permeability by Janus kinase (JAK)/signal transducer of the activators of transcription (STAT) signaling pathway (Marrero et al. 1995, Cheng et al. 2020, Perrotta et al. 2020). Moreover, ACE2 is also capable of converting Ang-I into Ang-(1–7), which leads to anti-fibrotic and anti-inflammatory effects in endothelial cells binding MAS receptors (Cheng et al. 2020). In addition, ACE2 expression may be inhibited by IL-6, IL-1β, and IFN-γ, changing the balance of Ang-2/Ang-(1–7) in favor of inflammation and vascular permeability (Kuba et al. 2005, de Lang et al. 2006).

In COVID-19 patients, high levels of a number of cytokines, including IL-6, IL-1β, tumor necrosis factor α (TNF-α) and IFN-γ, have been reported (Huang et al. 2020, McGonagle et al. 2020). The systemic outcome due to this effect is known as cytokine release syndrome (CRS) or as ‘cytokine storm’ and is believed to be a major cause of tissue damage in the pathophysiology of the disease (Ye et al. 2020). CRS is characterized by an overactive immune response that results in an excessive systemic increase in pro-inflammatory cytokines in response to a stimulus, in this case the viral infection (Behrens and Koretzky 2017).

Cytokine storm caused by SARS-CoV-2 infection can activate several signaling pathways in infected cells. Elevated levels of certain cytokines; IL-6, IL-1β and IFN-γ, are important activators of the JAK/STAT pathway (O'Shea et al. 2015, Murakami et al. 2019), activators of the mitogen-activated protein kinases (MAPKs) cascade (Mizutani et al. 2004, Grimes and Grimes 2020), and is also able to induce NF-κB signaling (Catanzaro et al. 2020, Jose and Manuel 2020).

### COVID-19: Symptoms and transmission

Although COVID-19 symptoms are similar to those seen in other coronavirus infections, they can vary, ranging from mild symptoms to severe illness (Grant et al. 2020). The most common symptoms include headache, loss of smell and taste, nasal congestion and runny nose, cough, muscle pain, sore throat, fever, diarrhea and breathing difficulties (Islam et al. 2020, Saniasiaya et al. 2020, Islam et al. 2021, Saniasiaya et al. 2021). Surprisingly, people having the same infection may have completely different symptoms, which can change over time. It has been shown that most people (around 81%) develop mild to moderate symptoms, while 14% develop severe symptoms and 5% suffer critical symptoms, leading to respiratory failure or multiorgan dysfunction. One third of virus-infected people do not develop noticeable symptoms at any time (Nogrady 2020, Oran and Topol 2021). It appears that all ages of the population are susceptible to SARS-CoV-2 infection, although clinical manifestations differ with age (Guan et al. 2020). Risk factors that predispose people towards the development of severe or critical COVID-19 include advanced age, male sex, obesity, diabetes and immunodeficiency. The signs of the diseases are seen after an incubation period of 1–14 days, most commonly around 3–5 days depending on the virus variant, and dyspnea and pneumonia develop within a median time of 8 days from illness onset.

Five common clusters of symptoms have been described for COVID-19 (Battagello et al. 2020): respiratory symptom cluster with cough, sputum, shortness of breath and fever leading to acute respiratory distress syndrome (ARDS); a musculoskeletal cluster with muscle and joint pain and fatigue; a neurological symptom cluster with headache, acute cerebral diseases, impaired consciousness, seizures, smell/taste impairment, muscle injury and neuralgia as the result of both peripheral and central nervous system failure; cardiovascular symptom cluster with myocardial injuries; renal system symptoms cluster with kidney failures and digestive symptom cluster with abdominal pain, vomiting and diarrhea (Cheng et al. 2004, Madjid et al. 2020, Mao et al. 2020, Siordia 2020, Wang et al. 2020a). The risk of death among individuals infected with COVID-19 can be calculated from the infection fatality risk (IFP) factor and has been estimated for 0.6%, as it may vary due to the age between 0.3% to 1.3% (Nishiura et al. 2020).

SARS-CoV-2 cell tropism may explain COVID-19 pathogenicity (Valyaeva et al. 2022). Nasal epithelial cells has been found to be very susceptible to viral infection, since they express highest levels of viral entry factors among all studied cells in the respiratory tract. Although lung tissue damage is a key feature of COVID-19 and the highest viral load were found in patients’ lungs, SARS-CoV-2 entry factors were expressed only in a small fraction of alveolar type 2 (AT2) cells. In addition, SARS-CoV-2 has also been found to infect ocular tissues (corneal and limbal cells), heart (cardiomyocytes and interstitial cells), gastrointestinal tract (enterocytes), pancreas (α and β cells), kidney, olfactory epithelium (nonneuronal cell types), and brain (neurons and glia) (Valyaeva et al. 2022).

The levels of viral pathogenicity are often associated with the levels of transmissibility, and experience shows that the higher the pathogenicity of a virus, the lower its transmissibility (Chen 2020). SARS-CoV-2 has a relatively low pathogenicity compared with emerging viruses like Ebola virus, SARS-CoV and MERS-CoV, and moderate transmissibility (Chen 2020). However, more recent VOCs, such as Delta and Omicron, show much higher transmissibility and outcompeted previous strains (Jung et al. 2022). SARS-CoV-2 is transmitted mainly through the respiratory route after an infected person coughs, sneezes, sings, talks or simply breathes. A new infection occurs when respiratory droplets or aerosols get into the mouth, nose or eyes of the people who are in close contact with the infected person (Nissen et al. 2020, Miller et al. 2021). It has been estimated that one infected person can generally infect, on average between two and three other people, depending also on the variant of the virus. Moreover, people can remain infectious in moderate cases for 7–12 days, and even up to two weeks in severe cases.

Apart from direct contact transmission, SARS-CoV-2 can infect via touching a surface or object covered with the virus and then touching the mouth, nose or eyes, although this is not the main way that the virus spreads (Meyerowitz et al. 2021). Infection mostly occurs indoors as it has been shown that sunlight inactivates the virus (Bueckert et al. 2020). On surfaces, the amount of active/viable virus decreases with time; however, it has been detected on contaminated surfaces for periods ranging from hours to days, depending on environmental conditions and the type of surface (Riddell et al. 2020).

## Animal models of COVID-19

Animal models have been essential tools for uncovering the pathogenesis and therapeutic targets of many human diseases. They are used to demonstrate the efficacy and safety of therapeutic agents, as well as for providing essential information on routes of administration, pharmacokinetics and pharmacodynamics and to identify key mechanism driving pathology *in vivo*. Although COVID-19 has appeared suddenly and recently, research studies have already developed and implemented many animal models for deciphering the secrets of the disease and provided insights into the SARS-CoV-2 biology.

An animal model of human disease should replicate the type of disease as close as possible in immunocompetent animals with a challenge dose and with a suitable exposure route that can be administered in humans. Furthermore, while choosing the animal model, the genetic background of the animals as well as the availability of immunological reagents need to be considered. Although SARS-CoV-2 is of zoonotic origin (Andersen et al. 2020), there are several major factors that complicate study of this virus in model organisms that closely mimics the clinical manifestation of the disease. The first limitation is the lack of infectivity of clinical isolates of SARS-CoV-2 in some model species, including mouse and dog (Shi et al. 2020). Another limitation is the absence of a persistent infection, immunopathology, severe acute respiratory distress syndrome, and systemic complications which characterize COVID-19 clinically. Furthermore, SARS-CoV-2 mainly causes severe COVID-19 in older people with comorbidities (Wang et al. 2020b), which represents a serious problem when attempting to use young and immunologically naïve laboratory animals in COVID-19 testing. For all these reasons, as well as the sense of urgency, scientists face a challenging set of problems while developing animal models that may involve compromises being made. In this respect, Table 1 presents all the advantages and limitations of the animal models used to date for COVID-19 research.

**Table 1.** Advantages and limitations of animal models for COVID-19.

Animal Model	Advantages	Limitations
**Non-human primates**	- Close in phylogeny with humans- Natural COVID-19 infectivity- Adaptive and Innate immunity highly similar to those in humans (mucosal transmission)- COVID-19 lung pathology detected in young and old animals- SARS-CoV-2 replication detected- ARDS symptoms detected,- Presence passive immunity- Drug efficacy and vaccine candidate testing	- Limited numbers of animals- Expensive maintenance- Do not develop the acute lung injury- Study of mild to moderate COVID-19, not severe illness- Limited availability- Long breeding time- More advanced cognition presents additional ethical issues
**Ferret**	- Useful to study viral diseases especially caused by respiratory pathogens- Caught reflex and fever symptom- COVID-19 pathology with some symptoms similar to human- Useful to study viral transmission- Used for longitudinal studies of immune response- Drug efficacy and vaccine candidate testing	- Study of asymptomatic to mild COVID-19, not moderate or severe illness- No pulmonary virus replication- No serious lung infection and oedema
**Mink**	- Study of moderate to severe COVID-19- COVID-19 pathology with respiratory symptoms similar to human- Useful to study viral transmission	- Difficult to handle under laboratory conditions
**Cat**	- COVID-19 infectivity- Natural susceptibility for SARS-CoV-2- Useful to study viral transmission- COVID-19 pathology with symptoms similar to human- Possibility of severe COVID-19 study- Lethality with pulmonary oedema reported- Useful to study viral transmission	- Study of asymptomatic to moderate COVID-19, not severe illness- Difficult to handle in biosafety level-3 conditions- Not standard animal model- Aggression problems
**Hamster**	- Immune response similar to human- Useful for drugs and vaccine testing- High homology in ACE2 with human- Presence of some COVID-19 symptoms and lung pathology (lung inflammation and injury)- Useful to study viral transmission- Production of neutralizing antibodies- Cost effective maintenance	- Lung pathology recovery by 14 days post-infection- Study of mild COVID-19 not moderate or severe illness- Lack of research tools comparing with mouse model- Not widely used
**Mouse**	- COVID-19 pathologies in knock-in models- Useful for vaccine and therapeutics evaluation- Useful for virus entry studies- Production of neutralizing antibodies- Wide range of research tools available- Immune response highly characterized- High throughput comparing with non-human primates	- Lack of natural COVID-19 infectivity- Insufficient affinity of murine ACE2 and SARS-CoV-2 S protein- Need of transgenic expression of hACE2 or viral adaptation is required- Non physiological conditions of the SARS-CoV-2 infection- CNS affected by neuro-invasion, lethal encephalitis- hACE2 tissue distribution and expression levels in knock-in mice- Differences in susceptibility for SARS-CoV-2 based on gender and age- Study of mild to moderate COVID-19, not severe illness- Limited availability- Relatively long breeding time
**Bat**	- They are SARS-CoV-2 originating species- Immune system evolution to the level to tolerate persistent viral infections- Viral detection and replication	- Difficult to handle under laboratory conditions- Not standard animal model- Lack of research tools comparing with other animal models
**Chicken**		- No susceptibility for SARS-CoV-2- No viral replication- Lack of symptoms or clinical signs of the disease
**Pig**		- No susceptibility for SARS-CoV-2- No viral replication- Lack of symptoms or clinical signs of the disease
**Dog**	- Possible susceptibility to SARS-CoV-2 but in very mild degree- Antibody testing possible	- Low susceptibility to infection with SARS-CoV-2- Not standard animal model
**Zebrafish**	- Low maintenance cost- Short lifespan- High fecundity levels, high throughput- Ease of manipulation, full-length or fragments of S protein injection- High homology of genes with human genome- Viral replication detected in swim bladder infection model- Presence of COVID-19 pathology despite the relatively low affinity between zebrafish Ace2 and viral S protein- Ease modeling of cytokine storm syndrome, hemorrhages, immune cells recruitment and emergency hematopoiesis after injection of viral proteins into the hindbrain- Ease of expression of human and viral proteins *in vivo*- Wide range of research tools available	- Lack of natural COVID-19 infectivity- Insufficient affinity of zebrafish Ace2 and SARS-CoV-2 S protein- Lack of lung- Growing temperature (28 vs. 37 °C)- Probably need of transgenic expression of hACE2 or viral adaptation is required- Non physiological conditions of the SARS-CoV-2 infection

### Ferrets and minks

**Figure 2.**
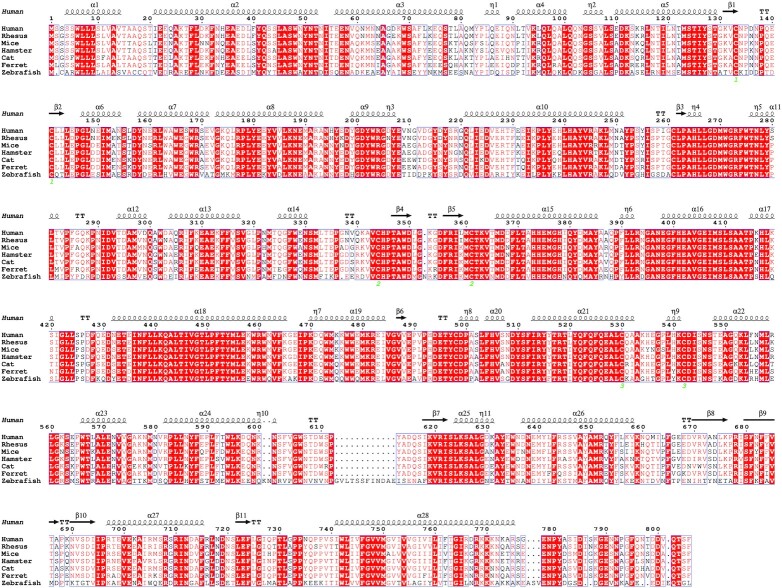
Multiple sequence alignment of different ACE2 sequences. Sequences of human ACE2 (Q9BYF1), rhesus macaque (F7AH40), mice (Q8R0I0), hamster (A0A1U7QTA1), cat (Q56H28), European domestic ferret (Q2WG88), and zebrafish (E7F9E5) were obtained from UniProt, aligned using CLUTAL OMEGA and represented using ESPript (Gouet et al. 1999). Strictly conserved residues have a red background. Symbols above the human sequence blocks represent secondary structure, springs represent helices and arrows represent β-strands.

Ferrets (*Mustela putorius furo*) have previously been used for the study of viral diseases especially for respiratory pathogens such as influenza. In addition, unlike mice and rats, ferrets exhibit a cough reflex and the domains of ACE2 involved in the interaction with RBD are well conserved (Fig. 2). Ferrets that were inoculated with the virus, were found to develop similar symptoms to those seen in humans within 2 and 12 days post-infection: namely an increase in body temperature suggestive of pyresis, reduced activity and appetite, and coughing (Kim et al. 2020, Shi et al. 2020). Histologically, the lungs of infected animals exhibited severe pulmonary lymphoplasmacytic perivasculitis and vasculitis 13 days post-infection (Kim et al. 2020). Moreover, it was found that SARS-CoV-2 was efficiently transmitted via direct contact and via the air (via respiratory droplets and/or aerosols) between ferrets (Richard et al. 2020). Naïve ferrets were placed in direct or indirect contact with inoculated ferrets (Kim et al. 2020). The study showed that all naïve ferrets placed in direct contact with infected animals displayed symptoms of infections 2–6 days post infection, whereas ferrets placed in indirect contact with inoculated animals, using a separation that allowed air to move, did not show any symptoms. Surprisingly, some of these asymptomatic ferrets tested positive, pointing to airborne transmission. In the same study, the lung histology of inoculated ferrets showed only mild signs of inflammation 4 days post infection. Ferrets have also been used for longitudinal studies of immune responses to infection with SARS-CoV-2. Intranasal inoculation with the virus and repeated measurements of upper respiratory tract gene transcripts from nasal washes showed a lower magnitude of upper airway immune responses than found for influenza A infection, as well as the induction of unique SARS-CoV-2 genes enriched for cell death and leukocyte activation-associated transcripts (Blanco-Melo et al. 2020). What is important, however, is that this study did not report any lower respiratory or systemic pathological findings. The lack of demonstrated pulmonary replication and edema in ferrets infected with SARS-CoV-2 might be regarded as a major limitation of these animals for the study of lung pathology. However, considering the similarity in the developed symptoms and transmissibility, ferrets can be a suitable model for disease transmission studies and pharmacology as well as to study Omicron VOC that mainly replicates in the upper respiratory tract (Karim and Karim 2021).

The relevance of ferret research is even clearer since an outbreak of SARS-CoV-2 infections with respiratory symptoms was reported in minks (*Mustela lutreola*), which also belongs to the Mustilidae family, in two farms in the Netherlands (Oreshkova et al. 2020). In contrast to ferrets, mink displayed moderate respiratory signs, including labored breathing and some of them died because of the infection. SARS-CoV-2 virus was detected in most of the throat and rectal swabs collected from dead mink. Like ferrets, the viral loads quantified in mink were higher in the throat than in rectal swabs. Although, *a priori*, mink may represent a suitable model for moderate-to-severe COVID-19, these animals are difficult to handle under laboratory conditions (Muñoz-Fontela et al. 2020).

### Cats

Several domestic cats (*Felis catus*), as well as zoo-housed big cats such as lions, tigers, pumas, cougars or snow leopards (the Felidae family), have tested positive for SARS-CoV-2 after showing signs of illness. The most probable cause is that they become ill after being exposed to an animal caretaker with COVID-19, despite the staff wearing personal protective equipment and following COVID-19 precautions. Although cats are not widely used to study respiratory diseases, the susceptibility they show for SARS-CoV-2 and the well conservations of ACE2 domains involved in the interaction with RBD (Fig. 2) make them a good model to study the viral infection *in vivo* and its transmission (Halfmann et al. 2020). However, cats are difficult to handle in biosafety level-3 conditions and do not show severe illness.

Transmission studies of SARS-CoV-2 have shown that two pairs (one inoculated and one naïve in each pair) of subadult (aged 6–9 months) and three pairs of juvenile cats (aged 10–14 weeks) that were housed together were infected. Fecal samples were analyzed for the presence of viral RNA, the result showing that in both groups only one out of the three naïve cats had viral RNA that could be detected in the fecal samples. This suggests a degree of transmission, although more limited than in ferrets. Unfortunately, scientists were unable to perform nasal washes on the subadult cats due to their aggression. It should be mentioned that one inoculated juvenile cat died 3 days post infection during this study, which may have been a result of severe disease in a young animal. On autopsy, histology of the lung tissues showed pronounced alveolar flooding, suggesting the development of pulmonary edema (Halfmann et al. 2020).

Moreover, a cat from a family with several relatives affected by COVID-19 developed severe respiratory clinical signs, suffering from severe respiratory distress and thrombocytopenia. The cat prompted humanitarian euthanasia and a detailed postmortem investigation was performed to assess whether SARS-CoV-2 was causing this condition. The results showed that the animal suffered from feline hypertrophic cardiomyopathy and severe pulmonary edema and thrombosis. Virus RNA was detected in a nasal swab, nasal turbinates, and mesenteric lymph node, but there was no evidence of histopathological lesions compatible with a viral infection (Segalés et al. 2020). Since the susceptibility of domestic cats has now been established, there is a need for extensive studies on these animals to ascertain the role of this species in the COVID-19 pandemic. Moreover, since severe disease can be reproduced in cats it may be useful for severe COVID-19 efficacy testing or for advances in veterinary medicine.

### Hamsters

Golden hamster (*Mesocricetus auratus*) has been used as an animal model to study human-associated diseases for over 60 years. Its immune responses to infectious pathogens are like those of humans and, as such, this model has many advantages for studying the pathogenesis of infection, including viruses, and for assessing the efficacy and interactions of medications and vaccines. Interestingly, the region of hamster and human ACE2 responsible for binding the RBD of Spike show a relatively high degree of homology (Chan et al. 2020b) (Fig. 2). When Golden Syrian hamster was inoculated intranasally with SARS-CoV-2, viral replication was detected in the lungs of infected hamsters together with lesions of pulmonary edema, inflammation, and cell death as assessed histologically. Infected hamsters were characterized by loss of weight and increased respiratory rates.

Moreover, infected animals were able to spread the virus to co-housed hamsters, suggesting its usefulness as a model for studying viral transmission. Interestingly, the infected co-housed hamsters showed similar signs of lung pathology but did not lose weight, which seems to suggest that the disease severity of inoculated hamsters is greater due to a higher amount of virus delivered to lungs. The early inflammatory response from lung samples showed high levels of IFNs and IL-6 (Chan et al. 2020b). Infected hamsters have been shown to generate neutralizing antibody responses after SARS-CoV-2 infection (Sia et al. 2020). Fourteen days post-infection, the lung pathology resolved in the hamster model. Taking all the above into consideration, these data suggest that hamsters might be very useful for modelling mechanisms of COVID-19 and for studying viral transmission.

### Mice

Mouse (*Mus musculus*) is one of the best animal models used to represent human diseases. Scientists have implemented also it to study COVID-19. However, the S proteins of SARS-CoV-2 are thought to have insufficient affinity for the murine ACE2 receptor for infection (Wan et al. 2020). Several key residues involved in the interaction with RBD are not conserved, such as Asp^30^ and Met^82^ (Fig. 2). Therefore, clinical isolates of SARS-CoV-2 have been adapted by serial passage in the respiratory tract of mice to produce related viruses that can cause similar symptoms, like marked infection in mouse lung, leading to interstitial pneumonia and inflammatory responses after intranasal inoculation (Gu et al. 2020). In another study, scientists developed a mouse model using inbred BALB/c mice whereby the interaction between S and mACE2 was remodeled. This remodeling resulted in a recombinant virus (SARS-CoV-2 MA) that used mACE2 for entry into BALB/c mice. The SARS-CoV-2 MA replicated well in adult and aged BALB/c mice, but causing more severe infections in the older animals (Dinnon et al. 2020).

To further overcome this affinity issue, scientists have generated a transgenic mouse K18-hACE2, whereby a transgene of human ACE2 (hACE2) expression is driven into mouse epithelial cells under the control of the human cytokeratin 18 (K18) promoter (McCray et al. 2007). Recently, it has been shown that SARS-CoV-2 can infect K18-hACE2 mice in a transgene-dependent manner (Bao et al. 2020). SARS-CoV-2 caused weight loss, evoked antibody responses, and developed lung inflammation, together with interstitial congestion, inflammatory exudate and epithelial damage (Bao et al. 2020, Hassan et al. 2020). Additional transgenic mouse strains have been developed to express human ACE2 using various promoters. HFH4-ACE2 transgenic mice were developed using the human *HFH4* promoter to express ACE2 in lung ciliated epithelial cell (Jiang et al. 2020), AC70 transgenic mice were developed using synthetic *CAG* promoter (a composite promoter consisting of the cytomegalovirus immediate-early enhancer and the chicken β-actin promoter and containing the rabbit globin splicing and polyadenylation site) to drive ubiquitous expression of ACE2 (Yoshikawa et al. 2009), and mouse *Ace2* promoter-driven human ACE2 transgenic mice were also generated (Yang et al. 2007, Bao et al. 2020). All the above ACE2 transgenic mice models can be used to study SARS-CoV-2 replication in the lung and its pathogenesis.

Most animal models, including mouse models, apart from their advantages, have their limitations. One of them is the lethal effect caused by neuro-invasion affecting CNS in K18-hACE2 mice after intranasal inoculation of SARS-CoV-2 (Kumari et al. 2021). Another issue is whether the tissue distribution and surface expression levels of hACE2 in hACE2 transgenic mice fully replicate those in humans. Murine ACE2 expression appears to be highly localized to the bronchial epithelium in mice (Sodhi et al. 2019), whereas hACE2 is more generally distributed in human lung (Hamming et al. 2004). Species differences in the distribution of ACE2 expression outside the lungs may also have implications for systemic responses to SARS-CoV-2 infection.

Apart from transgenic mice, an adeno-associated virus (AAV) delivery-based mouse model that expresses the SARS-CoV-2 receptor in the mouse lungs was developed to study SARS-CoV-2 pathogenesis (Gu et al. 2020, Hassan et al. 2020, Sun et al. 2020). In this model, artificially expressed hACE2 in non-relevant cell types in the mouse respiratory system by adenovirus transduction is making the pathology and immune response hard to interpret. However, such as model can be suitable for drug therapy and antibody testing. Therefore, although SARS-CoV-2 cannot infect wild type and laboratory inbred mouse strains, the animals can be used to study the development of neutralizing antibodies against the SARS-CoV-2 S protein, pseudo viral vaccine candidates, and antiviral drugs (Wang et al. 2020b).

Finally, several knockout mouse models have been developed in order to understand the pathology of SARS-CoV-2. Among them, *Ace*−/− knockout mice can be used to study the effects of ACE during acute lung injury (Imai et al. 2005) or *Tmprss2*−/−knockout mice to study the role of TMPRSS2 during the entry of SARS-CoV-2 into cells (Iwata-Yoshikawa et al. 2019).

### Non-human primates

Non-human primates (NHP) are close in phylogeny to humans and their ACE2 is highly conserved (Fig. 2), which make them a good model to study human diseases, and, in particular, COVID-19. To date, the NHP used for SARS-CoV-2 infections include rhesus macaques (*Macaca mulatta*) (Yu et al. 2020), cynomolgus monkeys (*Macaca fascicularis*) (Rockx et al. 2020), and common marmosets (*Callithrix jacchus*). For studies of SARS-CoV, African green monkeys (*Chlorocebus sabaeus*), squirrel monkeys (*Simia sciurea*) and moustached tamarins (*Saguinus mystax*) have also been used (Gong and Bao 2018). It was found that after SARS-CoV-2 infection rhesus macaques and cynomolgus monkeys developed the typical lung pathology lesions seen in humans. In addition, viral antigens were detected in alveolar epithelial cells and macrophages in these macaques (Lu et al. 2020b). In another study, the severity of interstitial pneumonia was compared between young and aged rhesus macaques, the severity being more pronounced in aged rhesus monkeys (Yu et al. 2020). Of particular note, after intratracheal inoculation of aged (around 15 years old) and young (3–5 years) animals with SARS-CoV-2, an age-related increase in viral load 7 days after inoculation was registered. Radiology and histology pointed to mild interstitial infiltrates in younger animals, whereas severe oedema, including alveolar flooding, was seen in aged macaques. Moreover, viral replication and shedding, along with COVID-19 symptoms with pathological lesions, were found in adult rhesus monkeys challenged with live virus (Munster et al. 2020). More specifically, when Rhesus macaques were inoculated intranasally, intratracheally, by mouth, and into both eyes with the virus, the presence of pulmonary infiltrates were observed radiologically, and oedema was measured gravimetrically. However, superficial inspection found lesions to be focal and sporadic. Moreover, in this case, a feature of ARDS that has rarely been seen in other models was alveolar flooding with the presence of hyaline membranes in lung histology (Matute-Bello et al. 2008). All the above was accompanied by an irregular breathing pattern and increased respiratory rate, suggesting hypoxemia. Finally, serum cytokine analysis detected no consistent evidence of systemic inflammation. Cynomolgus monkey is another NHP model that can be used to shed light into the mechanisms that drive severe COVID-19, as viral infection affected a greater proportion of the lungs in this species. Both young adult (4–5 years) and aged (15–20 years) cynomolgus monkeys were challenged with SARS-CoV-2 using a combined intranasal and intratracheal inoculation approach. Four days post infection two animals from each age group were autopsied, finding limited focal lesions in the lungs of animals from both groups. The lung lesions showed alveolar flooding and hyaline membrane formation, accompanied by other signs of diffuse alveolar damage co-localized with SARS-CoV-2 nucleocapsid staining (Le Bras 2020, Rockx et al. 2020).

Studies have also been carried out on rhesus monkeys to evaluate whether re-exposure to the virus can lead to the reoccurrence of the virus (Chandrashekar et al. 2020, Deng et al. 2020). Two animals were inoculated intratracheally with SARS-CoV-2 and then challenged again 28 days later. Lack of viral shedding after re-challenge in both macaques suggested development of protective immunity. However, this result should not be overinterpreted, as protective immunity wanes and reinfection rates have considerable increased during the Omicron wave (Flacco et al. 2022).

Apart from the infection studies carried out on NHP, the therapeutic efficacy of drugs (e.g. Remdesivir) and of many vaccine candidates have been studied in rhesus monkeys before undergoing testing in humans in clinical trials (Corbett et al. 2020, van Doremalen et al. 2020, Williamson et al. 2020).

Although NHP models can resemble human systems more closely than more phylogenetically distant animal models, it has to be remembered that these studies frequently use limited numbers of animals in the experiments (Curtis et al. 2018). Scientists have limited numbers of NHP available for the definitive proof of concept pathology studies, especially as some of the animals are needed for vaccine tests, so experimental designers are unfortunately forced to make compromises between the group size in early pathology studies and those for later preclinical trials of therapeutics.

### Other species

All the animal models presented in this work have been widely used in COVID-19 research laboratories during the ongoing pandemic. However, the list of the animal models is not necessarily complete, and scientists may well find better ones. It has been speculated that SARS-CoV-2 most probably originated from a coronavirus that infected bats (Andersen et al. 2020, Zhang et al. 2020). Lately, bats have become a particular research interest, although they are not commonly used as model organisms. Firstly, they appear to be the originating species of many zoonotic viruses and, secondly, their immune systems have evolved to the level that they can tolerate persistent infections with viruses of high virulence, which could accelerate viral evolution (Brook et al. 2020, Rabi et al. 2020). This suggests that wild bats would not be a good model, although several studies have been carried out on Egyptian fruit bats (*Rousettus aegyptiacus*) (Schlottau et al. 2020). In one such study, seven out of nine fruit bats intranasally infected with SARS-CoV-2 had a transient infection; the virus was detected in the nasal cavity of the animals, and they showed signs of rhinitis. In addition, SARS-CoV-2 was efficiently replicated in the upper respiratory tract and seroconversion was observed in seven out of nine of the bats. Viral RNA was also identified in the trachea, lung, and lung-associated lymphatic tissue in two animals that were euthanized at day 4 post infection.

The studies carried out on bat cell lines should be mentioned, as they demonstrate the mechanisms through which bat immune systems tolerate viral infections. The results showed decreased induction of NLRP3 inflammasomes (Ahn et al. 2019) and constitutive ubiquitous expression of antiviral IFN-ɑ (Zhou et al. 2020), suggesting that these mediators could be of potential interest for suppressing harmful inflammatory responses to viral SARS-CoV-2 infection or simply for reducing the viral load. In short, the intranasal infection of Egyptian fruit bat could reflect the reservoir host status and represent a useful model for studying SARS-CoV-2 infections.

Other species like chickens (*Gallus gallus domesticus*) or pigs (*Sus scrofa domesticus*) have also been tested for susceptibility to SARS-CoV-2. *In silico* data suggested that pigs and chickens or ducks might be susceptible to SARS-CoV-2 infection (Veljkovic et al. 2020). However, it was found that neither pigs nor chickens that were infected intranasally or oculo-oronasally were susceptible to SARS-CoV-2, and all swabs, organ samples, and contact animals were negative for viral RNA (Schlottau et al. 2020, Shi et al. 2020). Hence, unfortunately, no clear evidence of virus replication has been observed in pigs or chickens. Another study confirmed that piglets infected with SARS-CoV-2 via different routes, including intranasal, intratracheal, intramuscular and intravenous, were not susceptible to SARS‐CoV‐2. They lacked lesions and showed no presence of viral RNA in tissues or swabs, and no seroconversion was observed in pigs inoculated parenterally (Vergara-Alert et al. 2020). However, SARS-CoV-2 isolates have shown infectivity in rabbit and pig cells *in vitro* (Chu et al. 2020). Although *in vitro* does not always mean infection can occur *in vivo*, these model organisms may be useful for studies related to COVID-19.

Finally, dogs (*Canis lupus familiaris*) have been shown to be susceptible to SARS-CoV-2, but to a very mild degree. This was confirmed with two independent studies that concluded that dogs have a low susceptibility to infection with SARS-CoV-2 (Shi et al. 2020).

## Zebrafish models of COVID-19

**Figure 3.**
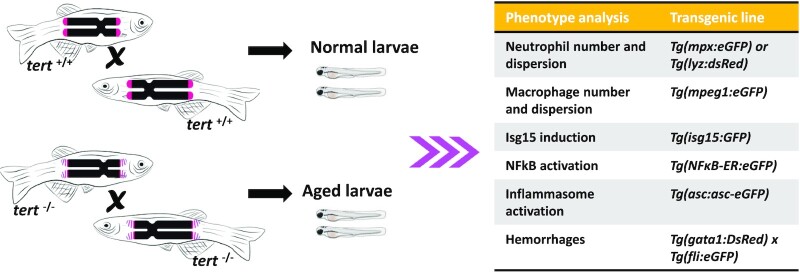
Analysis of COVID-19 vaccines and immunopathology in zebrafish larvae. Normal and aged larvae are obtained from wild type (wt, *tert*^+/+^) and telomerase-deficient (*tert*^−/−^) lines, respectively. Larvae of 2 days post-fertilization are then injected with recombinant spike variants or infected with SARS-COV-2, and several phenotypes analyzed with the indicated reporter lines at different times post-inoculation.

Zebrafish (*Danio rerio*) has been demonstrated as a reliable model system for studying human viral pathologies, including RNA viruses such as Chikungunya virus (Palha et al. 2013), and could potentially be regarded as suitable for rapid mass testing for COVID-19. However, there are no evidences supporting that coronaviruses infect zebrafish or other fish species. This vertebrate model has unique advantages, including transparent bodies, short lifespan, low maintenance costs, the easy production of large numbers of embryos and numerous reporter lines available to monitor immune and inflammatory responses (Fig. 3). Moreover, humans and zebrafish share 70% of the genome, while 84% of human genes known to be associated with human diseases have a counterpart in zebrafish: Hence, it deserves a special place in this review.

Comparative analysis of ACE2 molecules in vertebrates indicate that they are well conserved (Fig. 2). A single ortholog of mammalian ACE2 is present in zebrafish. However, the similarity and identity of zebrafish Ace2 amino acid motifs in the region involved in binding SARS-CoV-2 S protein is not as high as in macaques or ferrets (Fu et al. 2020) and several key residues involved in the interaction with RBD are not conserved, such as Asp^30^, His^34^ and Met^82^ (Fig. 2). Initially, to establish a mechanism in which zebrafish larvae could be infected with SARS-CoV-2, different methods of viral infection were used. For example, zebrafish larvae have been exposed to wild-type SARS-CoV-2 by bath immersion at 2 and 4 dpf (days post-fertilization). Unfortunately, 48 hours post-exposure, neither group had been infected and did not show signs of viral replication (Kraus et al. 2022, Laghi et al. 2022). This suggests that, despite the general conservation of ACE2 molecules in vertebrates, the differences in amino acids identified in zebrafish Ace2 are enough to prevent SARS-CoV-2 infectivity. Nevertheless, viral treatment caused a sustained inhibition of *ace2* expression, triggered type 1 cytokine response and inhibited type 2 cytokine response. In addition, at 3 dpf, the zebrafish larvae were microinjected across various sites (coelom, pericardium, brain ventricle, bloodstream, or yolk) with SARS-CoV-2, but this strategy also failed, and no infection or viral replication was observed. Finally, the microinjection into the swim bladder and coelomic cavity of 4 dpf larvae proved to be successful and, although no signs of disease were observed, RNA replication was detected after a brief period of degradation in the swim bladder but not the celomic cavity. Curiously, the swim bladder of zebrafish has been found to be a good model of human lung diseases (Zhang et al. 2016, Lee et al. 2019) and transcriptomic analysis has revealed the molecular homology between the swim bladder and the lung (Zheng et al. 2011).

Apart from the wild-type virus SARS-CoV-2 used for infection, some laboratories have used the recombinant RBD domain from S protein to study the host immune response. RBD of SARS-CoV-2 appears to mount innate immune responses that resemble the cytokine responses of mild COVID-19 patients. Recombinant SARS-CoV-2 S RBD was sufficient to cause olfactory, renal and cardiovascular pathologies in larvae and adult zebrafish (Ventura Fernandes et al. 2022). However, there is no evidence that this response is directly dependent on the SARS-CoV-2 Spike—zebrafish Ace2 interaction. Moreover, zebrafish injected with recombinant Spike protein—fragment 16 to 165 (rSpike), which corresponds to the N-terminal portion of the protein—produced an acquired and innate immune response and even showed adverse effects. Seven days after injection of rSpike into the adult zebrafish IgM antibodies were detected. The efficiency of this process increased on the 14th day, conferring a reduction in the mortality rate of the immunized animals and suggesting the similarity to infected human individuals with COVID-19 (Ventura Fernandes et al. 2022). Antibody production was found not only in the serum, but also in the zebrafish eggs as a result of passive transfer to protect the offspring. Furthermore, fish injected with rSpike produced a deleterious inflammatory response similar to severe cases of COVID-19 in humans. In addition, it was shown that rSpike injection affected the nervous system and caused significant renal and liver alterations and ovary damage in adult zebrafish (Ventura Fernandes et al. 2022).

To overcome the limitations of zebrafish model for COVID-19, a humanized zebrafish model has been generated by xeno-transplanting human alveolar epithelial cells (A549) intramuscularly in the vicinity of the lobe of the swim bladder in adult zebrafish. Seven-days post xeno-transplantation of human cells the fish were injected with full-length SARS-CoV-2 S protein. Using full-length S protein provides information as to whether other fragments of the protein, apart from the N- terminal fragment containing RBD domain, is responsible for the generation of the viral response. Higher mortality, behavioral fever, renal cell necrosis, skin hemorrhages and inflammation in the swim bladder were the main features of SARS-CoV-2 S protein induced pathologies, that can be easily used for the development of reliable therapies and drugs (Balkrishna et al. 2020).

Our group has very recently shown that S1 domain of recombinant S protein (S1WT) can provoke local and systemic inflammation, CRS, emergency myelopoiesis, strong recruitment of the immune cells to the site of the protein injection and intracranial hemorrhages *in vivo* in zebrafish larvae (Tyrkalska et al. 2022). Notably, the injection of S1WT into the hindbrain of 48 hours post fertilization (hpf) larvae strongly induced the production of pro-inflammatory cytokines, the production of neutrophils and macrophages in response to the threat, as well as caused hemorrhages in the head of 25% of the larvae. Pharmacological inhibition of the canonical inflammasome robustly alleviated S1 protein-induced inflammation and emergency myelopoiesis, and the administration of angiotensin (1–7) fully rescued S1-induced hyperinflammation and hemorrhages in this model. Additionally, this model has also allowed the study of various Spike variants showing big differences in their abilities to cause CRS as well as being recognized by the host immune system. In particular, S1γ protein was more proinflammatory and S1δ was less proinflammatory than S1WT and, strikingly, S1β promoted delayed and long-lasting inflammation (Tyrkalska et al. 2022). Further, the injection of full-length RNA of wild type S into one-cell stage embryos mimicked the phenotype of the larvae injected with the recombinant Spike proteins. Thus, the forced expression of full-length wild type S led to the induction of strong inflammation and generation of hemorrhages in the head of about 45% of the larvae, confirming the proinflammatory effects of wild type S in zebrafish larvae (Tyrkalska et al. 2022).

Finally, there seems to be a need for the generation of zebrafish expressing hACE2 in specific tissues and/or the generation of zebrafish-adapted SARS-CoV-2 strains. Such models could increase the efficiency of SARS-CoV-2 infection in more physiological conditions and show clinical symptoms or gross pathological changes similar to those seen in humans. The ubiquitous expression of hACE2 could provide important information about the result of the viral infection in context of the whole body, showing disorders or damage in all systems and organs. A model expressing hACE2 under a specific promoter, for example in olfactory neurons using the *ora4* promoter (Sepahi et al. 2019), would also be informative. As these cells are present in the olfactory organs of zebrafish, expressing the hACE2 receptor would mimic the physiological way of SARS-CoV-2 infection. It could give us an update about how the virus is entering via the nasal route. Based on all the available information gathered until now and all the possibilities that the zebrafish model provides, this animal could act as a novel vertebrate model to elucidate SARS-CoV-2 pathophysiology and to screen drugs and other therapies targeting COVID-19.

### Zebrafish models for the discovery of novel antivirals to treat COVID-19

The COVID-19 pandemic has revealed the lack of powerful antiviral compounds available that can be mobilized rapidly and made available for the treatment of re-emerging or emerging viral diseases (Adamson et al. 2021). Strategies to combat viral diseases are prevention through the administration of vaccines, or treatment using antiviral drugs and antibodies. Developing antiviral therapies requires a fundamental knowledge of the biology of the virus and, in particular, of its interaction with the host cell. As obligate parasites, viruses depend on the cellular processes of their host cells. Viruses, with their compact genomes and lack of cellular anatomy, offer many fewer druggable targets. However, despite this inconvenience, drug development strategies to deal with COVID-19 pandemic can be classified into the following groups: (i) blocking viral structural proteins, thus inhibiting virus–host interaction and therefore viral entry and/or capsid assembly; (ii) inhibiting the enzymes responsible for the synthesis and replication of viral RNA; (iii) targeting viral virulence factors that mediate their escape from the host's immune system; and (iv) targeting specific host receptors, such as ACE2 (Jamshaid et al. 2020, Dolgin 2021, Srivastava and Ahmad 2021). Taking into considerations all these targets, several treatments are in different stages of development and some have already been approved or recommended according to the patient's profile (Jamshaid et al. 2020, Dolgin 2021, Srivastava and Ahmad 2021). In this respect, the zebrafish is an excellent model in terms of human health and acts as a kind of bridge between *in vitro* assays and mammalian *in vivo* studies for evaluating the efficacy, appropriate dose, toxicity and the simultaneous assessment of different drugs (Ding et al. 2011, Cassar et al. 2020). Cell-based tests provide limited information on the absorption, distribution, metabolism, excretion, and toxicity of antiviral compounds, whereas zebrafish *in vivo* tests often reveal insights into these pharmacological aspects. It is worth pointing out that zebrafish larvae have functional livers, kidneys, and blood-brain barriers (Jeong et al. 2008, Goldstone et al. 2010, Li et al. 2011). To produce phenotypes *in vivo* in zebrafish assays, compounds must possess the ability to be absorbed, reach target tissue, and avoid rapid metabolism and excretion. In this way, compounds that are discovered in zebrafish can be rapidly tested in mammalian models *in vivo* with minimal optimization of pharmacological aspects.

**Figure 4.**
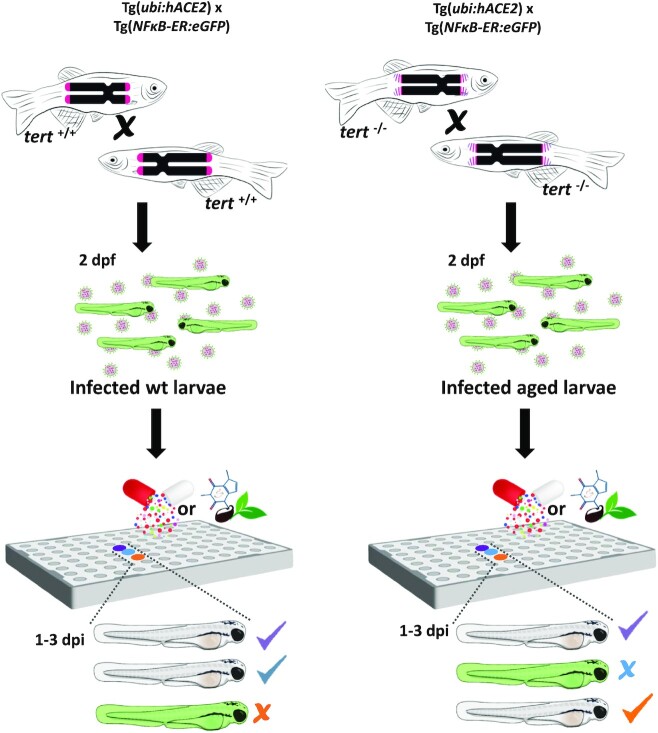
Chemical screening in zebrafish larvae to identify antiviral compounds to treat COVID-19. Normal and aged larvae are obtained from transgenic fish expressing hACE2 driven by the ubiquitin promoter [*Tg(ubi:hACE2*)] and an NF-κB reporter [*Tg(NFκB-ER:eGFP*)]. Larvae of 2 days post-fertilization (dpf) are then infected with SARS-COV-2, distributed in 96-well plates, treated with FDA-approved or natural chemical libraries, and NF-kB analyzed at 1–3 days-post-infection (dpi). Positive hits can reduce NF-kB activation, easily assayed as decreased green fluorescence. Three classes of compounds will be identified: group 1 reduce inflammation in normal and aged larvae (violet), group 2 reduce inflammation in normal larvae (blue), and group 3 reduce inflammation in aged larvae (orange).

While vaccines for COVID-19 have been developed in record-breaking time—they were approved within 12 months—the usual timing for the approval of a new antiviral therapeutic agent can exceed ten years. Therefore, the repositioning of known drugs that have already been Food and Drug Administration (FDA)-approved represents one of the most promising strategies for the identification and development of treatments for emerging infectious diseases, including COVID-19, considerably shortening the drug development time (Riva et al. 2020, Srivastava and Ahmad 2021). In this case, the path to clinical research is shortened because the identified compounds have already been approved for human use or at least have undergone prior pharmacokinetic analysis and safety testing. Screening natural products for antiviral activity is also a good approach, taking into consideration their biological relevance and diversity. The ‘COlleCtion of Open NatUral producTs’ (COCONUT) contains structures and sparse annotations for over 400,000 non-redundant natural products (Sorokina and Steinbeck 2020). For both approaches, the zebrafish as an animal model of many human diseases, has demonstrated its efficacy in the screening of repurposed drugs (Rovira et al. 2011, Cousin et al. 2014, Ganzen et al. 2021) and natural products (Liang et al. 2015, Brillatz et al. 2018, Cheng et al. 2018), and also for determining specific therapeutic targets in humans (Fig. 4).

### Zebrafish models for the study of COVID-19 vaccines

According to the World Health Organization (WHO), ‘vaccination is the administration of agent-specific, but safe, antigenic components that in vaccinated individuals can induce protective immunity against the corresponding infectious agent’ (https://www.who.int/travel-advice/vaccines. Accessed November 9, 2022). Minor side effects are common after vaccine administration and go away within a few days, including local reactions at the injection site such as pain, swelling, and erythema, and systemic reactions such as fever, irritability, headache, diarrhea or vomiting, tiredness, muscle or joint pains, drowsiness, and rash. More serious reactions have also been described although they are extremely rare, including seizures, permanent brain damage, encephalitis, meningitis, Guillain-Barré Syndrome, coma, pneumonia, life-threatening infections, among others. Moreover, as with any medicine, there is a very remote chance of a vaccine causing a severe allergic reaction, other serious injury, or death (Spencer et al. 2017) (Centers for Disease Control and Prevention. Possible side-effects from vaccines. http://www.cdc.gov/vaccines/vac-gen/side-effects.htm. Accessed November 9, 2022).

The efforts to produce effective vaccines against COVID-19 since the pandemic began, have been enormous, to the point that currently there are 323 vaccine candidates in different stages of development, including 99 undergoing clinical testing and 18 already in use (Table S1) (Tregoning et al. 2020, Shrotri et al. 2021). The different platforms used for the development of these vaccines are shown in Table S2. At the time of writing, COVID-19 vaccines from Pfizer-BioNTech (Polack et al. 2020), Moderna (Baden et al. 2021), and Johnson and Johnson (Sadoff et al. 2021) are the only three authorized by the FDA and administered in the U.S., while those same three, along with a fourth produced by Oxford-AstraZeneca (Voysey et al. 2021), and fifth Nuvaxovid produced by Novavax NVX-CoV2373 (Heath et al. 2021, Parums 2022) have been authorized by the European Medicines Agency (EMA) and are being administered in the European Union (Table S2). Adverse effects produced by these five vaccines do not seem to be more severe than those described for any other vaccine and, in the vast majority of cases, they are described as a combination of fever, headache, myalgia, and general malaise (Table S2) (Menni et al. 2021, Sprent and King 2021). These symptoms are present in ∼60% of recipients after the second dose of the vaccines, and it has been proposed that they can be attributed simply to the exacerbated production of type I IFN (Sprent and King 2021). Although clinical trials of vaccines involve thousands of people, they are not designed to detect extremely rare side effects that might appear in fewer than one case per 10,000 vaccinations. However, it is not surprising that those very rare events—such as severe allergic reactions or blood clots—appear in safety reports, since hundreds of millions of people are being vaccinated against COVID-19. Despite this, studies to analyze the rate of such rare adverse events in vaccinated populations compared with the probability that they occur by chance in unvaccinated people have not found any clear links beyond their coincidence in time. Anyway, public-health agencies keep track of potential side effects through reporting systems such as the World Health Organization's platform VigiBase, the EMA's EudraVigilance and the United States’ Vaccine Adverse Event Reporting System (Remmel 2021).

The development of novel tools and models that allow a rapid and in-depth study of the adverse effects of COVID-19 vaccines is essential for the safety of the world population, as the continuous emergence of new SARS-CoV-2 variants that might present resistance to the immunity generated by current vaccines could make it necessary to develop new vaccines in a very short period of time (Janik et al. 2021, Krause et al. 2021). In addition, the availability of detailed and reliable data on the side effects of vaccines is crucial to reinforce their credibility and to avoid mistrust of the population, which might fuel the anti-vaccine movements that are already increasing vaccine hesitancy in some communities. Although vaccines are one of the most important medical advances in history, their unquestionable success, paradoxically, has led to the unexpected issue that confidence in them has decreased among the population in recent years, since most vaccine-preventable illnesses have become unfamiliar to modern parents and, therefore, their risk perception is low (Spencer et al. 2017). Furthermore, another cause of distrust in vaccines is that they have been associated with multiple and serious side effects, and even though most such claims arise from anti-vaccine conspiracy theories and have never been demonstrated, they are counter-productive for the vaccination programs worldwide (Dubé et al. 2013, Jolley and Douglas 2014, Spencer et al. 2017).

The zebrafish presents unique characteristics that have made this small fish a very popular vertebrate model in biomedicine in recent years, including in the field of vaccination (Myllymäki et al. 2018, Bailone et al. 2020, Tyrkalska et al. 2021). This animal model offers researchers the possibility of treating individuals with different doses of exactly the same antigenic components that are present in the vaccines, and to monitor animals afterwards (Fig. 3). For this, dozens of variables can be measured and analyzed after administration of the vaccine, including survival experiments, gene expression assays, behavioral studies, the analysis of vascular damage and clots formation, the determination of cell numbers for the different blood cell populations, etc.

In conclusion, the zebrafish could be a powerful tool to characterize the potential adverse effects of vaccines against COVID-19 both rapidly and effectively. This could be extremely relevant from a biomedical point of view, since it may be necessary to urgently develop novel vaccines against any new SARS-CoV-2 variants that may arise. Besides that, the availability of clear and reliable information on possible adverse effects of vaccination against COVID-19 would help to counteract the increasing influence of anti-vaccine movements.

### Zebrafish models to help understand the relevance of aging in COVID-19

What has been clear since the start of the COVID-19 pandemic is that the main risk factor for mortality is age, with an infection fatality rate ranging between 8–36% in people over 80 years old (Docherty et al. 2020, Verity et al. 2020). The main molecular mechanism of aging that could be responsible for aggravating the SARS-CoV-2 infection is telomere shortening, which results in chromosome instability, replicative senescence and/or apoptosis (Blasco 2005, Lopez-Otin et al. 2013). Short telomeres are associated with the presence of fibrosis in lungs, liver and kidneys, intestinal atrophy and bone marrow aplasia, as a consequence of the loss of the regenerative capacity of these tissues (Armanios and Blackburn 2012). Pulmonary tissue is particularly important in SARS-CoV-2 infection and in the development of the disease. It has already been shown in several mouse models of pulmonary fibrosis that dysfunctional telomeres in AT2 cells are associated with the origin of this disease, due to the impairment of the stem cell function by induced senescence (Alder et al. 2015, Povedano et al. 2015). Several groups have recently demonstrated in two different patient cohorts that shorter telomeres are associated to increased severity of COVID-19 (Froidure et al. 2020, Sanchez-Vazquez et al. 2021). In addition to what occurs in the elderly, the mortality of COVID-19 is also higher in adults with cardio-metabolic diseases and in men, a shorter telomere length (TL) being the common factor in these groups (Aviv and Shay 2018). According to these results, short TL correlates with severity, and TL has been proposed as a prognostic factor of COVID-19 (Froidure et al. 2020, Sanchez-Vazquez et al. 2021): As a consequence, and it is speculated that telomerase activation therapies could ameliorate fibrosis-like pathologies in COVID-19 patients (Bernardes de Jesus et al. 2012, Martinez and Blasco 2017, Povedano et al. 2018).

Apart from its impact on proliferation and regeneration, several studies have also demonstrated that short TL directly impact the immune cell function. Telomerase, the reverse transcriptase that maintains telomeres (Blackburn and Collins 2011), is active in hematopoietic stem cells, but this activity is insufficient to prevent age-associated telomere shortening and ultimately leads to cellular senescence, culminating in the cessation of replication. This phenomenon, known as immunosenescence, is a process associated with lymphopenia, the progressive depletion of naïve T cells and the reduced proliferation ability of those cells (Wherry and Kurachi 2015), accompanied by the development of a low-grade inflammatory status in many elderly people known as inflammaging (Franceschi et al. 2000). It has been suggested that both, immunosenescence and inflammaging are risk factors for severe COVID-19 in older people (Cunha et al. 2020, Pietrobon et al. 2020). A recent report shows that abnormally short telomeres in patients with telomere-related gene mutations are sufficient to drive T cell aging (Wagner et al. 2018). As a result of immunosenescence, it has been reported that adults with shorter telomeres have impaired immune surveillance against persistent viral infections (Fulop et al. 2013) and, are more sensitive to experimentally-induced respiratory viral infection (Cohen et al. 2013). In addition, short TL of leukocytes is associated with more severe acute respiratory distress syndrome and worse survival in patients with sepsis (Liu et al. 2020). Severe lymphopenia is a characteristic feature not only in COVID-19 (Diao *et al*. 2020, Zheng *et al*. 2020) but also in SARS and MERS (Booth et al. 2003, Min et al. 2016). The inverse correlation between leukocyte TL and the severity of disease has been mentioned (Benetos et al. 2021, Dos Santos et al. 2021), and lymphocyte subset counts have been proposed as novel diagnostic and prognostic biomarkers for COVID-19 (Jin et al. 2021, Wu et al. 2021, Zahran et al. 2021). Repurposing metformin, a biguanide that attenuates multiple hallmarks of aging, including lowering telomere attrition and senescence (Kulkarni et al. 2020), has been proposed as a useful treatment for COVID-19 infection (Omarjee et al. 2020).

The versatility of the zebrafish model is that it also covers the study of the aging process (Kishi 2004). Hence, several zebrafish models have been developed to study the main hallmarks of aging. First, the telomerase-deficient zebrafish model (*tert*-/-) shows tumor protein 53 (Tp53)-dependent premature aging (life span 1.5 vs. 3 years of wild type animals) because of telomere shortening and chromosome instability (Anchelin et al. 2013, Henriques et al. 2013). Importantly, premature aging is observed in the first generation, representing a great advantage over mouse models, and it also shows anticipation, as observed in patients (Anchelin et al. 2013). More recently, the recombination-activating gene 1-deficient zebrafish line (*rag1*-/-), which lacks adaptive immunity, also has been reported as a model for studying inmunosenescence as a consequence of oxidative stress and chronic inflammation (Novoa et al. 2019). Although both lines offer an excellent opportunity for modeling the impact of telomere length, (immuno)senescence and aging in COVID-19, the Tert-deficient line, in particular, could be used in high-throughput drug screening (Patton et al. 2021), since aged larvae with short telomeres can be obtained already in the second generation (Anchelin et al. 2013) and the impact of the compounds can be easily analyzed (Figures 3 and 4). These studies will hopefully allow the identification of novel drugs or the repurposing of those already available for improving the antiviral response or mitigating the CRS of the elderly, the most vulnerable group to SARS-CoV-2 infection.

## Concluding remarks and future directions

There is an urgent need for novel animal models to study SARS-CoV-2 infection, for the evaluation of vaccine mechanisms of action and side-effects, and for the identification of novel antiviral drugs to treat the most susceptible people, such as the elderly. The unique advantages of the zebrafish, which combines the advantages of both vertebrate and invertebrate models, make this animal model a promising complement to those already available. To leverage the powerful advantages of this model, it is required to generate lines that can be infected by SARS-CoV-2, for example by expressing hACE2 ubiquitously or in specific tissues of interest. We firmly believe that the zebrafish model will help to elucidate SARS-CoV-2 pathophysiology and will allow novel therapies and treatments targeting COVID-19 to be identified and developed.

## Supplementary Material

fuac042_Supplemental_File
