# Supplementary material for: Zebrafish models of COVID-19
Source: FEMS Microbiol Rev. 2022 Nov 2;47(1):fuac042. doi: 10.1093/femsre/fuac042 (PMC9841970; doi:10.1093/femsre/fuac042)
Supplement: fuac042_Supplemental_File [file fuac042_supplemental_file.pdf]

Table S1. COVID-19 vaccines under consideration by type and stage of development. See https://vac-lshtm.shinyapps.io/ncov_vaccine_landscape/ for updates (Shrotri *et al.*, 2021). Accessed July, 2022.

	Stage of development							
Vaccine type	Pre-clinical	Phase I	Phase I/II	Phase II	Phase III	Phase IV	Total	In use
RNA	25	14	6	5	3	3	56	2
DNA	16	7	5	-	2	-	30	1
Non-replicating viral vector	24	7	4	1	2	4	42	5
Replicating viral vector	20	-	1	-	1	-	22	-
Inactivated	11	6	3	-	6	3	29	8
Live attenuated	2	1	-	-	1	-	4	-
Protein subunit	76	15	11	6	19	1	128	5
Virus-like particle	19	1	2	1	2	-	25	-
Other/Unknown	4	1	1	1	-	-	7	-
Total	197	52	33	14	36	11	343	21

Table S2. COVID-19 vaccines approved for their use in the European Union and the United States of America up to date (Centers for Disease Control and Prevention (CDC), https://www.cdc.gov/coronavirus/2019-ncov/vaccines/different-vaccines.html; Scotland's national health information service (NHS inform, https://www.nhsinform.scot/covid-19-vaccine/the-vaccines/the-vaccines-used-to-protect-against-coronavirus; European Medicines Agency (EMA), https://www.ema.europa.eu/en/human-regulatory/overview/public-health-threats/coronavirus-disease-covid-19/treatments-vaccines/covid-19-vaccines); (Tregoning *et al.*, 2020, Sokolowska *et al.*, 2021). Very common, more than 1 in 10 people; common, up to 1 in 10 people; uncommon, up to 1 in 100 people; rare, up to 1 in 1,000 people; very rare, up to 1 in 10,000 people; not known, cannot be estimated from the available data. Websites accessed July, 2022.

	VACCINE				
MANUFACTURER	Pfizer, Inc., and BioNTech	ModernaTX, Inc.	AstraZeneca	Janssen Pharmaceuticals Companies of Johnson & Johnson	Novavax CZ a.s.
NAME	BNT162b2, Comirnaty	mRNA-1273, Spikevax	ChAdOx1-S, Vaxzevria	JNJ-78436735, Ad26.COV2.S	Nuvaxovid NVX-CoV2373
TYPE OF VACCINE	mRNA	mRNA	Non-replicating viral vector	Non-replicating viral vector	SARS-CoV-2 recombinant spike protein
AGE	5 years and older	12 years and older	18 years and older	18 years and older	18 years and older
ADMINISTRATION	Two doses injected in the muscle of the upper arm (21 days apart)	Two doses injected in the muscle of the upper arm (28 days apart)	Two doses injected in the muscle of the upper arm (4-12 weeks apart)	One dose injected in the muscle of the upper arm	Two doses injected in the muscle of the upper arm (21 days apart)
EFFICACY	95% (86% in 65 years and older)	94.1% (86% in 65 and older)	70% (100% for severe disease)	72% (86% against severe disease)	90.4% (88.9% in 65 years and older)
INGREDIENTS	<u>Active ingredient</u>: nucleoside-modified mRNA encoding the viral spike (S) glycoprotein of SARS-CoV-2<br><br><u>Inactive ingredients</u>: 2[(polyethylene glycol (PEG))-2000]-N,N-ditetradecylacetamide; 1,2-distearoyl-sn-glycero-3-phosphocholine; cholesterol; (4-hydroxybutyl)azanediyl)bis(hexane-6,1-diyl)bis(2-	<u>Active ingredient</u>: nucleoside-modified mRNA encoding the viral spike (S) glycoprotein of SARS-CoV-2<br><br><u>Inactive ingredients</u>: PEG2000-DMG: 1,2-dimyristoyl-rac-glycerol, methoxypolyethylene glycol; 1,2-distearoyl-sn-glycero-3-phosphocholine; cholesterol; SM-102: heptadecan-9-	<u>Active ingredient</u>: recombinant, replication-deficient chimpanzee adenovirus vector encoding the SARS-CoV-2 Spike (S) glycoprotein<br><br><u>Inactive ingredients</u>: L-histidine; L-histidine hydrochloride monohydrate; magnesium chloride hexahydrate; polysorbate 80; ethanol; sucrose;	<u>Active ingredient</u>: recombinant, replication-incompetent Ad26 vector, encoding a stabilized variant of the SARS-CoV-2 Spike (S) protein<br><br><u>Inactive ingredients</u>: polysorbate-80; 2-hydroxypropyl-β-cyclodextrin; citric acid monohydrate; trisodium citrate dihydrate;	<u>Active ingredient</u>: SARS-CoV-2 spike protein<br><br><u>Inactive ingredient</u>: Matrix-M adjuvant with Fraction-A (42.5 micrograms) and Fraction-C (7.5 micrograms) of *Quillaja saponaria*, Disodium hydrogen phosphate heptahydrate, Sodium dihydrogen phosphate monohydrate,

	hexyldecanoate); sodium chloride; monobasic potassium phosphate; potassium chloride; dibasic sodium phosphate dihydrate; sucrose	yl 8-((2-hydroxyethyl)(6-oxo-6-(undecyloxy) hexyl) amino) octanoate; tromethamine; tromethamine hydrochloride; acetic acid; sodium acetate; sucrose	sodium chloride; disodium edetate dehydrate; water for injections	sodium chloride; ethanol	Disodium hydrogen phosphate dihydrate, Sodium chloride, Polysorbate 80, Cholesterol, Phosphatidylcholine (including all-rac-α-Tocopherol), Potassium dihydrogen phosphate, Potassium chloride, Sodium hydroxide (for the adjustment of pH), Hydrochloric acid (for the adjustment of pH), Water for Injections
SIDE EFFECTS DESCRIBED AND THEIR FREQUENCY	<u>Common</u>: redness at injection site, nausea, vomiting<br><br><u>Uncommon</u>: enlarged lymph nodes, feeling unwell, arm pain, insomnia, injection site itching, allergic reactions such as rash or itching<br><br><u>Rare</u>: temporary one sided facial drooping, allergic reactions such as hives or swelling of the face<br><br><u>Not known</u>: severe allergic reaction inflammation of the heart	<u>Very common</u>: pain and swelling at the injection site, tiredness, chills, fever, swollen or tender lymph nodes under the arm, headache, muscle and joint pain, nausea and vomiting<br><br><u>Common</u>: redness, hives and rash at the injection site<br><br><u>Uncommon</u>: itching at the injection site<br><br><u>Rare</u>: swelling of the face, which may affect people who had facial cosmetic injections in	<u>Very common</u>: tenderness, pain, warmth, itching or bruising where the injection is given, generally feeling unwell, feeling tired (fatigue), chills or feeling feverish, headache, feeling sick (nausea), joint pain or muscle ache<br><br><u>Common</u>: swelling, redness or a lump at the injection site, fever (≥38°C), being sick (vomiting or diarrhoea), pain in legs or arms, flu-like	<u>Very common</u>: pain at the injection site, headache, tiredness, muscle pain and nausea<br><br><u>Common</u>: coughing, joint pain, fever, chills and redness, swelling at the injection site<br><br><u>Uncommon</u>: sneezing, tremor, throat pain, rash, sweating, muscle weakness, pain in the arms and legs, backache, weakness, feeling generally unwell	<u>Very common</u>: headache, feeling sick (nausea) or getting sick (vomiting), muscle ache, joint pain, tenderness or pain where the injection is given, feeling very tired (fatigue), generally feeling unwell<br><br><u>Common</u>: redness where the injection is given, swelling where the injection is given, fever (>38°C), chills, pain or discomfort in the arm, hand, leg and/or foot

	(myocarditis or pericarditis)	the past, and weakness in muscles on one side of face (acute peripheral facial paralysis or palsy)	symptoms, such as high temperature, sore throat, runny nose, cough and chills<br><br>Uncommon: sleepiness or feeling dizzy, decreased appetite, abdominal pain, enlarged lymph nodes, excessive sweating, itchy skin, rash or hives<br><br>Very rare: following widespread use of the vaccine there have been extremely rare reports of blood clots in combination with low level of blood platelets. When these blood clots do occur, they may be in unusual or atypical locations (e.g. brain, liver, bowel, spleen)<br><br>Not known: severe allergic reaction (anaphylaxis), rapid swelling under the skin in areas such as the face, lips, mouth and throat, which may cause difficulty in swallowing or breathing (angioedema),	Rare: hypersensitivity (allergy) and itchy rash	(pain in the extremity)<br><br>Uncommon: enlarged lymph nodes, high blood pressure, itchy skin, rash or hives, redness of the skin, itchy skin where the injection is given<br><br>Allergic reaction: feeling faint or light-headed, changes in your heartbeat, shortness of breath, wheezing, swelling of your lips, face, or throat hives, rash nausea or vomiting or stomach pain

			low blood platelets (thrombocytopenia), hypersensitivity		
EFFICACY AND SAFETY PUBLICATION	(Polack *et al.*, 2020)	(Baden *et al.*, 2021)	(Voysey *et al.*, 2021)	(Sadoff *et al.*, 2021)	(Heath *et al.*, 2021)

References

Baden LR, El Sahly HM, Essink B, *et al.* (2021) Efficacy and Safety of the mRNA-1273 SARS-CoV-2 Vaccine. *N Engl J Med* **384**: 403-416.

Heath PT, Galiza EP, Baxter DN, *et al.* (2021) Safety and Efficacy of NVX-CoV2373 Covid-19 Vaccine. *N Engl J Med* **385**: 1172-1183.

Polack FP, Thomas SJ, Kitchin N, *et al.* (2020) Safety and Efficacy of the BNT162b2 mRNA Covid-19 Vaccine. *N Engl J Med* **383**: 2603-2615.

Sadoff J, Gray G, Vandebosch A, *et al.* (2021) Safety and Efficacy of Single-Dose Ad26.COV2.S Vaccine against Covid-19. *N Engl J Med* **384**: 2187-2201.

Shrotri M, Swinnen T, Kampmann B & Parker EPK (2021) An interactive website tracking COVID-19 vaccine development. *Lancet Glob Health* **9**: e590-e592.

Sokolowska M, Eiwegger T, Ollert M, *et al.* (2021) EAACI statement on the diagnosis, management and prevention of severe allergic reactions to COVID-19 vaccines. *Allergy* **76**: 1629-1639.

Tregoning JS, Brown ES, Cheeseman HM, Flight KE, Higham SL, Lemm NM, Pierce BF, Stirling DC, Wang Z & Pollock KM (2020) Vaccines for COVID-19. *Clin Exp Immunol* **202**: 162-192.

Voysey M, Clemens SAC, Madhi SA, *et al.* (2021) Safety and efficacy of the ChAdOx1 nCoV-19 vaccine (AZD1222) against SARS-CoV-2: an interim analysis of four randomised controlled trials in Brazil, South Africa, and the UK. *Lancet* **397**: 99-111.
